# The prevalence of hyperglycemia and its association with perioperative outcomes in gynecologic surgery: a retrospective cohort study

**DOI:** 10.1186/s13741-023-00307-1

**Published:** 2023-06-02

**Authors:** Katherine F. Chaves, Joseph R. Panza, Mutiya A. Olorunfemi, Christine M. Helou, Annie N. Apple, Zhiguo Zhao, Laura L. Sorabella, Susan D. Dumas, Rony A. Adam, Lauren S. Prescott

**Affiliations:** 1 Division of Gynecology, Department of Obstetrics and Gynecology, Vanderbilt University Medical Center, Nashville, TN 37212 USA; 2 Division of Female Pelvic Medicine and Reconstructive Surgery, Department of Obstetrics and Gynecology, Vanderbilt University Medical Center, Nashville, TN USA; 3 Meharry Medical College, Nashville, TN USA; 4 Vanderbilt University School of Medicine, Nashville, TN USA; 5 Department of Biostatistics, Vanderbilt University Medical Center, Nashville, TN USA; 6 Division of Obstetric Anesthesiology, Department of Anesthesiology, Vanderbilt University Medical Center, Nashville, TN USA; 7 Division of Gynecologic Oncology, Department of Obstetrics and Gynecology, Vanderbilt University Medical Center, Nashville, TN USA

**Keywords:** Complications, Diabetes, Gynecologic surgery, Hyperglycemia

## Abstract

**Background:**

Preoperative hyperglycemia has been associated with perioperative morbidity in general surgery patients. Additionally, preoperative hyperglycemia may indicate underlying impaired glucose metabolism. Thus, identification of preoperative hyperglycemia may provide an opportunity to mitigate both short-term surgical and long-term health risk. We aimed to study this phenomenon specifically in the gynecologic surgery population. Specifically, we aimed to evaluate the association between preoperative hyperglycemia and perioperative complications in gynecologic surgery patients and to characterize adherence to diabetes screening guidelines.

**Methods:**

This retrospective cohort study included 913 women undergoing major gynecologic surgery on an enhanced recovery pathway from January 2018 to July 2019. The main exposure was day of surgery glucose ≥ 140 g/dL. Multivariate regression identified risk factors for hyperglycemia and composite and wound-specific complications.

**Results:**

Sixty-seven (7.3%) patients were hyperglycemic. Diabetes (*aOR* 24.0, 95% *CI* 12.3–46.9, *P* < .001) and malignancy (*aOR* 2.3, 95% *CI* 1.2–4.5, *P* = .01) were associated with hyperglycemia. Hyperglycemia was not associated with increased odds of composite perioperative (*aOR* 1.3, 95% *CI* 0.7–2.4, *P* = 0.49) or wound-specific complications (*aOR* 1.1, 95% *CI* 0.7–1.5, *P* = 0.76). Of nondiabetic patients, 391/779 (50%) met the USPSTF criteria for diabetes screening; 117 (30%) had documented screening in the preceding 3 years. Of the 274 unscreened patients, 94 (34%) had day of surgery glucose levels suggestive of impaired glucose metabolism (glucose ≥ 100 g/dL).

**Conclusion:**

In our study cohort, the prevalence of hyperglycemia was low and was not associated with higher risk of composite or wound-specific complications. However, adherence to diabetes screening guidelines was poor. Future studies should aim to develop a preoperative blood glucose testing strategy that balances the low utility of universal glucose screening with the benefit of diagnosing impaired glucose metabolism in at-risk individuals.

**Supplementary Information:**

The online version contains supplementary material available at 10.1186/s13741-023-00307-1.

## Background

The deleterious effects of postoperative hyperglycemia include increased complication rates, length of stay, and mortality, but the impact of preoperative hyperglycemia is less clear (Frisch et al. 2010; Thourani et al. 1999). An emerging body of evidence demonstrates that preoperative hyperglycemia, independent of diabetes status, is associated with an elevated risk of surgical site infections and mortality (Abdelmalak et al. 2014; Frisch et al. 2010; Jackson et al. 2012; Noordzij et al. 2007). However, these studies included mostly general surgery and/or male patients.

The lack of research examining the prevalence and effect of preoperative hyperglycemia in exclusively female or gynecologic surgery patients make it difficult to formulate preoperative glucose testing guidelines. In the gynecologic oncology population, the Enhanced Recovery After Surgery Society® recommends optimization of postoperative glucose in hyperglycemic patients, but they do not discuss preoperative glucose testing (Nelson et al. 2016). Additionally, as of 2019, the United States Preventive Services Task Force (USPSTF) advises diabetes screening in general for adults age 40–70 who are overweight or obese, but current preoperative testing algorithms make no formal glucose screening recommendations (Fleisher et al. 2007; Siu 2015). Notably, in the general preoperative setting, the prevalence of new diabetes diagnoses ranges from 1.2 to 10% (Abdelmalak et al. 2010; Grek et al. 2009; Hatzakorzian et al. 2011). Thus, identification of preoperative hyperglycemia may provide an important opportunity to mitigate both short-term surgical and long-term health risk.

Our primary objective was to estimate the prevalence of preoperative hyperglycemia in patients undergoing gynecologic surgery and evaluate its associations with perioperative complications and wound morbidity. The secondary objectives included identifying the risk factors for hyperglycemia and characterizing adherence to USPSTF diabetes screening guidelines.

## Materials and methods

This retrospective cohort study, conducted at a tertiary academic medical institution, included women age 18 and older who underwent gynecologic surgery on the Vanderbilt Department of Obstetrics and Gynecology enhanced recovery pathway (ERP) from January 2018 through July 2019. Exclusion criteria included age < 18 years old, multispecialty cases led by a non-gynecologic primary surgeon, and unplanned or emergency surgery. The Vanderbilt Institutional Review Board approved this study (IRB 171,143) prior to data collection under its quality improvement/non-research determination as the ERP was implemented on a departmental level for quality improvement purposes. Thus, informed consent was not required.

The Vanderbilt University Medical Center (VUMC) instituted the gynecologic ERP in January 2018 for patients undergoing hysterectomies, exploratory laparotomies, and complex urogynecologic procedures. This pathway, which is derived from the Enhanced Recovery After Surgery Society® pathway, includes preoperative patient optimization, patient education, and multimodal pain control (Ljungqvist et al. 2017; Nelson et al. 2016). Additionally, patients can consume clear liquids until 2 (nondiabetics) or 4 (diabetics) h prior to surgery, and nondiabetic patients receive an approximately 9-g liquid carbohydrate load 2 h prior to surgery. As this pathway includes optimal glucose control, all patients are to receive a blood glucose measurement in the preoperative holding area on the day of surgery. Hyperglycemic patients are treated with insulin according to an institutional sliding-scale algorithm with a target blood glucose of < 140 mg/dL. Our institution does not have a policy regarding elective case cancellation based on hyperglycemia the day of surgery. The shared decision to proceed with surgery is made with the patient, surgeon, and anesthesiologist after weighing risks and benefits.

Enhanced recovery pathway patient data were prospectively recorded by multiple authors (K. C., J. P., M. O., C. H.) using Research Electronic Data Capture (REDCap) tools hosted at VUMC (Harris et al. 2019, 2009). The accuracy and completeness of the database were optimized by utilizing the validation and required fields tools in REDCap and through review meetings with the project leader (L. P.) aimed at discussing and confirming unexpected values or trends.

Demographic and clinical data captured included age, race, ethnicity, body mass index (BMI), insurance status, medical comorbidities, the American Society of Anesthesiologists (ASA) class, and smoking status. Perioperative characteristics recorded included most recent hemoglobin within 30 days of surgery, timing of each case (first of the day or not first), whether or not the patient had preoperative outpatient ERP nursing and anesthesia visits, surgical approach (laparoscopic/robotic, laparotomy, or vaginal), departmental division that completed the surgery (general gynecology, gynecologic oncology, urogynecology, or minimally invasive gynecologic surgery (MIGS)), operative time, and estimated blood loss (EBL). Additionally, we noted whether each nondiabetic patient met USPSTF criteria for diabetes screening (herein referred to as “nondiabetic at-risk patients”) (Selph et al. 2015).

The main exposure was clinically actionable preoperative hyperglycemia, defined as a blood glucose ≥ 140 g/dL on the day of surgery. We based this threshold on guidelines from Duggan, Klopman, and Berry who recommend continued blood glucose monitoring in patients with a blood glucose at this threshold on the day of surgery (Duggan et al. 2016). Although our nondiabetic patients receive a 9-g carbohydrate load 2 h prior to surgery, a recent study in gynecologic oncology patients showed that the median preoperative glucose after a much higher 50-g load was 122 mg/dL (Alimena et al. 2020). Additionally, a blood glucose ≥ 140 g/dL after a 75-g carbohydrate load defines impaired glucose tolerance (American Diabetes Association 2019; Siu 2015). Thus, although the majority of our patients are not strictly fasting the morning of surgery because of this carbohydrate drink, they receive a relatively small carbohydrate load, and we felt this threshold remained clinically relevant.

The primary aim was to estimate the prevalence of actionable hyperglycemia and evaluate its association with 30-day composite perioperative complication and 30-day wound complication. Our secondary aims were to evaluate risk factors for hyperglycemia and adherence to USPSTF diabetes screening guidelines. Hyperglycemia is associated with multiple physiologic aberrations, including alterations in inflammatory, coagulation, and hormonal pathways (Duggan et al. 2016). As such, we assessed for a wide range of complications: the composite binary perioperative complication metric was defined as the presence of at least one intraoperative or postoperative complication which included intra- or postoperative blood transfusion, venous thromboembolism, and complication of any of the following organ systems: respiratory (pneumonia, pulmonary embolism, reintubation, pneumothorax, other); cardiovascular (arrhythmia, congestive heart failure, myocardial infarction, cardiac arrest or requiring cardiopulmonary resuscitation, other); gastrointestinal (ileus, diarrhea, constipation, obstruction, anastamotic leak, other); central nervous system, renal (acute kidney injury, urinary tract infection, urinary retention, other); endocrine; or infectious (sepsis). Thirty-day wound complication included surgical site infection and/or wound disruption. Surgical site infection was defined as a clinically diagnosed infection of the surgical incision or organ space within thirty days of surgery. Wound disruption included superficial disruption (reopening of superficial wound layers) and dehiscence (reopening of the entire wound thickness). We defined diabetes screening compliance as a recorded hemoglobin A1C, fasting blood glucose, or oral glucose tolerance test (in the VUMC electronic health record or in available scanned outside medical records) within 3 years prior to surgery for non-diabetic at-risk patients (Siu 2015). In unscreened patients, we noted if patients had a blood glucose level 100–125 mg/dL (if fasting, the definition of impaired fasting glucose) or ≥ 126 mg/dL (if fasting, diagnostic of type 2 diabetes mellitus) (Siu 2015). The dataset supporting the conclusion of this article is included within the article’s additional files (Additional file 1).

Patients’ demographic characteristics, comorbidities, and perioperative characteristics were summarized with median and interquartile range (IQR, continuous variables) or frequency and percentage (categorical variables) by hyperglycemia status. Differences between groups were assessed using Wilcoxon rank-sum or Pearson’s chi-squared tests. To evaluate the association between hyperglycemia with composite complications and wound complications, both univariate and multivariable logistic regressions were used, and the odds ratios (ORs) with 95% confidence intervals (CIs) were reported as the effect measurements. In all adjusted models, patients age (continuous, linear), BMI (continuous, linear), ASA class (1–2 vs. ≥ 3), diabetes (yes vs. no), cardiovascular comorbidity (yes vs. no), malignancy (yes vs. no), surgical approach (laparotomy vs. minimally invasive), division (gynecologic oncology vs. urogynecology vs. generalist/MIGS), operative time (continuous, linear), and EBL (continuous, linear) were adjusted. For all continuous variables in the models, restricted cubic splines were initially considered, and then, a chunk test on all nonlinear terms were conducted. In the final models, only linear terms were included, based on the nonsignificant chunk test result. In the exploratory analyses, univariate logistic regressions were used, and no multivariable modeling was attempted due to the limited frequencies of complications and large number of potential risk factors. In a post hoc power analysis, we estimated the minimal detectable OR, at 80% study power and 5% type 1 error rate, of 30-day composite perioperative complication rates between hyperglycemic and non-hyperglycemic patients using the *Z*-test with pooled variance. Two-sided *P*-values less than 0.05 were considered statistically significant. All analyses were conducted with R software version 4.2.

## Results

A total of 1111 gynecologic patients were identified in the ERP REDCap database during the time period of interest. Twenty of these patients were excluded because they had a non-gynecologic primary surgeon, and 178 were excluded because they did not have a preoperative blood glucose measurement documented, yielding a total of 913 patients included in the analysis. Compared with the patients analyzed, the 178 patients who were excluded due to a lack of documented blood glucose were less likely to be diabetic (4% vs. 15%, *P* < 0.001) but were otherwise similar in all other documented characteristics. Similarly, excluded patients had a similar rate of complications as included patients (31% vs 26%, *P* = 0.16).

Patient demographics and clinical factors are listed in Table 1. Hyperglycemic patients were older (median age 60 vs. 49 years, *P* < 0.001) and had a higher BMI (median 32 vs. 29 kg/m^2^, *P* = 0.007). No statistically significant differences were observed in race, ethnicity, smoking status, and insurance status between the two groups. With regard to comorbidities, a significantly greater proportion of hyperglycemic patients had a malignancy (39% vs. 16%, *P* < 0.001), cardiovascular disease (78% vs. 47%, *P* < 0.001), diabetes mellitus (76% vs. 10%, *P* < 0.001), and an ASA class ≥ 3 (82% vs. 55%, *P* < 0.001). Regarding the perioperative characteristics, the two groups were similar with regard to proportion of cases that were the first scheduled cases of the day, preoperative nurse teaching and anesthesia visits, surgical approaches, preoperative hemoglobin levels, and EBL. A greater proportion of hyperglycemic patients as compared to non-hyperglycemic patients underwent surgery with gynecologic oncology as opposed to benign divisions (52% vs. 22%, *P* < 0.001). Additionally, they underwent significantly longer surgeries (median 183 vs. 143 min, *P* = 0.01).

**Table 1:** Patient demographics, comorbidities, and perioperative characteristics by hyperglycemia status^a^

Patient characteristics	Hyperglycemia		*P*^*b*^
	**No** ***(n* = *846)***	**Yes** ***(n* = *67)***	
**Demographic characteristics**			
**Age, years**	49.0 (41.0, 63.0)	60.0 (47.0, 68.5)	< .001
**BMI, kg/m**^**2**^	29.2 (25.5, 34.9)	32.3 (26.9, 39.2)	.007
**Race**			0.89
White	676 (79.9%)	54 (80.6%)	
Non-white	170 (20.1%)	13 (19.4%)	
**Ethnicity**			0.45
Hispanic	788 (93.1%)	64 (95.5%)	
Non-Hispanic	58 (6.9%)	3 (4.5%)	
**Smoking**			0.37
Never	574 (67.8%)	48 (71.6%)	
Current	82 (9.7%)	3 (4.5%)	
Former	190 (22.5%)	16 (23.9%)	
**Insurance**			0.57
Private	457 (54.0%)	33 (49.3%)	
Public	314 (37.1%)	30 (44.8%)	
Other	70 (8.3%)	4 (6.0%)	
Missing	5 (0.6%)	0 (0%)	
**Comorbidities**			
**ASA class**			< .001
I–II	379 (44.8%)	12 (17.9%)	
≥ III	467 (55.2%)	55 (82.1%)	
**Malignancy**			< .001
No	704 (83.2%)	41 (61.2%)	
Yes	139 (16.4%)	26 (38.8%)	
Missing	3 (0.4%)		
**Cardiovascular disease**			< .001
No	449 (53.1%)	15 (22.4%)	
Yes	397 (46.9%)	52 (77.6%)	
**Respiratory disease**			0.17
No	703 (83.1%)	60 (89.6%)	
Yes	143 (16.9%)	7 (10.5%)	
**Diabetes mellitus**			< .001
No	763 (90.2%)	16 (23.9%)	
Yes	83 (9.8%)	51 (76.1%)	
**Perioperative characteristics**			
**Case timing**			0.59
First	407 (48.1%)	29 (43.3%)	
Not first	439 (51.3%)	38 (56.7%)	
Missing	5 (0.6%)	0 (0%)	
**Preoperative nurse visit**			0.91
No	83 (9.8%)	15 (22.4%)	
Yes	761 (90.0%)	60 (89.6%)	
Missing	2 (0.2%)	0 (0%)	
**Preoperative anesthesia visit**			0.36
No	33 (3.9%)	5 (7.5%)	
Yes	812 (96.0%)	62 (92.5%)	
Missing	1 (0.1%)	0 (0%)	
**Surgical approach**			0.15
MIS	659 (77.9%)	47 (70.1%)	
Laparotomy	187 (22.1%)	20 (29.9%)	
**Division**			< .001
General Gyn/MIGS	384 (45.4%)	15 (22.4%)	
Urogynecology	275 (32.5%)	17 (25.4%)	
Gynecologic oncology	187 (22.1%)	35 (52.2%)	
**Operative time, minutes**	143.0 (107.0, 207.0)	183.0 (119.0, 240.0)	.01
**Upper quartile EBL, mL**	100.0 (50.0, 217.5)	100.0 (50.0, 225.0)	0.76

*ASA* American Society of Anesthesiologists, *BMI* Body mass index, *Hgb* Hemoglobin, *EBL* Estimated blood loss, *MIGS* Minimally gynecologic invasive surgery

^a^Data reported are median (interquartile range) for continuous variables and *n* (%) for categorical variables. ^b^Determined by Wilcoxon rank sum for continuous variables and Pearson’s chi-squared test for categorical variables

The overall prevalence of hyperglycemia was 7.3% (67 out of 913). The prevalence of hyperglycemia was significantly greater in the 134 patients with diabetes compared to the 779 patients without diabetes (*n* = 51 (38%) vs. *n* = 16 (2%); *P* < 0.001 respectively, Table 2).

**Table 2:** Prevalence of hyperglycemia by diabetic status^a^

	**Non-diabetics** ***(n* = *779)***	**Diabetics** ***(n* = *134)***	***P***^***c***^
**Blood glucose category**^**b**^			< .001
Not hyperglycemic	763 (97.9%)	83 (61.9%)	
Hyperglycemic	16 (2.1%)	51 (38.1%)	

^a^Data reported as *n* (%). ^b^Hyperglycemic defined as blood glucose ≥ 140 g/dL. ^c^As determined by Pearson’s chi-squared test

A total of 217 (26%) non-hyperglycemic patients experienced perioperative complications compared to 25 (37%) hyperglycemic patients (*P* = 0.04, Additional file 2 — Supplemental Table S1). There were 43 (5%) wound complications in non-hyperglycemic patients and 6 (9%) in hyperglycemic patients (*P* = 0.17).

In the univariate analysis, hyperglycemia was associated with an increased risk of composite complication (*OR* 1.7, 95% *CI* 1.0–2.9, *P* = 0.04, Additional file 2 — Supplemental Table S2). However, after controlling for key potential clinical and demographic risk factors (see “Materials and methods”), the association was no longer statistically significant (*OR* 1.3, 95% *CI* 0.7–2.4, *P* = 0.49, Table 3). (Our post hoc power calculation demonstrated that we achieved 80% power to detect an odds ratio of 2.3.) Hyperglycemia was not associated with wound complications in univariate or multivariable analysis (*OR* 1.1, 95% *CI* 0.7–1.5, *P* = 0.76 in multivariable analysis (Table 3).

**Table 3:** Multivariable adjusted associations with composite and wound complications^a^

	**Composite complication** ***(events* = *242)***		**Wound complication** ***(events* = *49)***	
**Patient characteristics**	**OR (95% *CI)***	***P***	**OR (95% *CI*)**	***P***
Hyperglycemia^b^	1.26 (0.65–2.43)	0.49	1.06 (0.7–0.51)	0.76
Age, years^c^	1.35 (0.94–1.95)	0.11	0.97 (0.7–1.35)	0.88
BMI, kg/m^2^	1.05 (0.84–1.32)	0.65	1.09 (0.86–1.39)	0.48
Operative time^c^	1.48 (1.19–1.85)	< 0.001	1.11 (0.88–1.39)	0.38
Upper quartile EBL	1.29 (1.13–1.47)	< 0.001	1.00 (0.97–1.03)	0.89
ASA ≥ III	1.11 (0.76-–.61)	0.60	1.09 (0.79–1.51)	0.63
Malignancy	1.18 (0.65–2.16)	0.58	1.09 (0.78–1.53)	0.62
Cardiovascular disease	1.00 (0.68–1.47)	1.00	1.09 (0.79–1.51)	0.610
Diabetes	1.14 (0.68–1.91)	0.61	1.09 (0.78–1.54)	0.61
Surgical approach (laparotomy vs. MIS)	2.13 (1.39–3.27)	< 0.001	1.28 (0.92–1.79)	0.14
Division				14
General Gyn/MIGS	Reference		Reference	14
Urogynecology	1.12 (0.7–1.78)	0.64	0.97 (0.69–1.36)	0.85
Gynecology	0.86 (0.45–1.62)	0.63	1.09 (0.77–1.53)	0.63

*ASA* American Society of Anesthesiologists, *BMI* Body mass index, *EBL* Estimated blood loss, *MIGS* Division of Minimally Invasive Gynecologic Surgery, *MIS* Minimally invasive surgical route (i.e., laparoscopic, robotic, or vaginal surgery)

^a^Results were estimated from multivariable logistic regression models. For wound complication, to account for the limited number of events, penalized maximum likelihood estimation was used. More details are available in the “Materials and methods” section. ^b^Hyperglycemia defined as a blood glucose ≥ 140 g/dL. ^c^ For continuous variables, OR reflect comparison of third quartile to first quartile

Although hyperglycemia was the main exposure as opposed to an outcome in this study, as a secondary objective, we aimed to identify risk factors for hyperglycemia due the potential utility of using these risk factors in preoperative glucose testing algorithms. In multivariable analysis adjusted for age, BMI, ASA class and cardiovascular disease, diabetes mellitus (*OR* 24.0, 95% *CI* 12.3–46.9, *P* < 0.001), and malignancy (*OR* 2.3, 95% *CI* 1.2–4.5, *P* = 0.01) were associated with increased odds of preoperative hyperglycemia (Table 4).

**Table 4:** Multivariable adjusted associations with hyperglycemia

	**Hyperglycemia**^**a**^^**,**^ ***(events* = *67)***	
**Patient characteristics**	**OR (95% *CI)***	***p***
Age, years^b^	1.23 (0.7–2.16)	0.47
BMI, kg/m^2b^	0.92 (0.64–1.32)	0.66
ASA ≥ 3 ^b^	1.37 (0.63–2.97)	0.42
Malignancy	2.32 (1.21–4.46)	.01
Cardiovascular disease	1.14 (0.54–2.42)	0.72
Diabetes	23.99 (12.26–46.92)	< . 001

*ASA* American Society of Anesthesiologists. *BMI* Body mass index

^a^Hyperglycemia defined as a blood glucose ≥ 140 g/dL. ^b^For continuous variables, *OR* reflect comparison of third quartile to first quartile

With regard to adherence to USPSTF guidelines, 391 of 779 (50%) nondiabetic patients met USPSTF criteria for diabetes screening. Of these, 117 (30%) had documented diabetes screening in the 3 years preceding surgery. Of the 274 unscreened patients, 94 (34%) had day of surgery glucose levels suggestive of impaired glucose metabolism. (79 patients had a blood glucose 100–125 mg/dL, and 15 patients had a blood glucose ≥ 126 mg/dL — the thresholds, if fasting, to diagnose impaired fasting glucose and type 2 diabetes mellitus (Siu 2015)).

## Discussion

The overall prevalence of hyperglycemia in this population was low at 7.3%, with the majority being diabetic. Only 2.1% of nondiabetic patients were hyperglycemic the day of their surgery, lower than previous studies reporting a prevalence around 20% (Bochicchio et al. 2005; Jackson et al. 2012). However, these previous studies included almost entirely men, identified male sex as a risk factor, or contained patients whose surgical indication (i.e., trauma or colon cancer) is associated with hyperglycemia. Thus, the fact that all gynecologic patients are biological females and that many are undergoing scheduled surgery for benign conditions, it likely places our population at inherently lower risk of hyperglycemia than previously studied populations.

Additionally, we did not find evidence that hyperglycemia was associated with composite perioperative complications. This is in contrast to the work of others that has shown increased morbidity in surgical patients with preoperative hyperglycemia (Frisch et al. 2010; Jackson et al. 2012; Noordzij et al. 2007; Wang et al. 2014). Frisch et al. and Noordzij et al. both demonstrated the increased odds of mortality in patients with preoperative hyperglycemia; however, the percentage of low-risk procedures (which, according to the American College of Cardiology/American Heart Association (ACC/AHA) classification used by both studies, includes gynecologic surgeries) ranged from 2 to 8% (Fleisher et al. 2007; Frisch et al. 2010; Noordzij et al. 2007). Frisch et al. included high-risk surgical subspecialities including neurosurgery and vascular surgery, while the majority of cases captured by Noordzij et al. were considered intermediate-high risk. Additionally, these comparator studies included male patients, which comprised half of patients in the case of Frisch et al. and the majority of patients in Noordzij et al. Thus, the fact that our study included exclusively gynecologic (ACC/AHA low-risk) surgeries and female patients may explain the lack of association.

Our post hoc power calculation demonstrated that our study was powered to detect an odds ratio of 2.3 for composite complication in hyperglycemic vs non-hyperglycemic patients. It is challenging to make direct comparisons because Noordzij et al. and Frisch et al. both use mortality as their outcome (an outcome that seemed inappropriate for our patient population), but the effect size in both studies was roughly 1.7–2.0 at levels of hyperglycemia similar to ours. Thus, we may have been underpowered to detect the smaller differences in complications found in our study (*OR* 1.7).

Similarly, studies in the general surgery population have demonstrated association between preoperative hyperglycemia and wound complications, while we did not (Jackson et al. 2012; Wang et al. 2014). Most (78%) of our patients underwent minimally invasive surgery, and the overall prevalence of wound complications was low (5%). Thus, our study may have been underpowered to study this outcome. Notably, however, a recent study by Alimena et al. also did not demonstrate an increased risk of infectious complications in hyperglycemic patients undergoing laparotomy for gynecologic malignancy (Alimena et al. 2020).

Fifty percent of our nondiabetic patients met 2015 USPSTF criteria for diabetes screening (Siu 2015). This is substantially higher than in the primary care population, where approximately 25% of patients meet criteria (O’Brien et al. 2016). Additionally, adherence to USPSTF diabetes screening guidelines was poor; among those who met criteria for diabetes screening, only 30% had a documented screening on file in the 3 years preceding surgery. This is lower than in the primary care population, where adherence with screening is reported around 50% (Bullard et al. 2015). Additionally, 34% of our unscreened patients had blood glucose levels suggestive of impaired glucose metabolism (although we could not formally diagnose impaired fasting glucose or diabetes because our patients are not strictly fasting the day of surgery as they receive a small carbohydrate load.) These findings highlight the unrealized opportunity to provide primary care in the preoperative setting. Although current preoperative testing guidelines do not make recommendations regarding diabetes screening, many advocate that providers take advantage of the perioperative time to screen for this prevalent, and often undiagnosed, disease (Cowie et al. 2009; Fleisher et al. 2007; Grek et al. 2009; Sheehy and Gabbay 2009; Wang et al. 2014).

Strengths of our study include the utilization of a prospectively built database with a diverse cohort of patients including patients undergoing procedures performed by both generalist and specialized surgeons. Thus, information gleaned from this work is applicable to other gynecology departments as a whole. With regard to limitations, our patients largely underwent minimally invasive surgery, which is inherently associated with fewer complications than open surgery, and wound complication rates were low. Hence, our study may have been underpowered to assess differences in these outcomes. Additionally, we used a relatively low threshold (140 mg/dL) to define hyperglycemia, and few patients had severe hyperglycemia (e.g., 19/913 had a blood glucose greater than 200 mg/dL). This may also have limited our ability to detect a difference in complications between the two groups.

With regard to diabetes screening, the USPSTF and the American Diabetes Association (ADA) have different guidelines (American Diabetes Association 2019; Siu 2015). The ADA recommends screening in patients with a BMI greater than 25 kg/m^2^ and at least one of nine known risk factors, which include information about family history, lifestyle, and medical history. Due to concerns about accurately characterizing patient risk factors retrospectively, we chose to utilize the USPSTF screening guidelines, which rely only on age and BMI. Hence, it is possible that we missed patients whose physicians followed and were compliant with ADA recommendations, as we only sought out diabetes screening data in patients who met USPSTF criteria. However, a large model utilizing a national database predicted that 27 million more Americans would be screened by using ADA as opposed to USPSTF criteria (Dall et al. 2014). Hence, it is most likely that, had we employed ADA criteria, we would have identified many more patients requiring screening, and our compliance rates would be even lower.

## Conclusion

The prevalence of actionable hyperglycemia in gynecologic surgery patients at our institution over a 17-month period was low. Aside from diabetes, malignancy was the only significant risk factor for preoperative hyperglycemia. Additionally, in our cohort, hyperglycemic patients were not at higher risk of composite or wound-specific perioperative complications. However, adherence to national diabetes screening guidelines was poor, a finding that highlights the unrealized opportunity presented in the preoperative period to identify individuals with undiagnosed diabetes. Future studies should aim to develop a preoperative blood glucose testing strategy that balances the low utility of universal glucose checks in nondiabetics without malignancy with the benefit of diagnosing impaired glucose metabolism in at-risk individuals.

## Supplementary Information

**Additional file 1.**

**Additional file 2: Supplemental Table S1.** Summary of complications, overall and by hyperglycemia status. **Supplemental Table S2.** Associations estimated from univariate analysis.

## Data Availability

All data generated or analyzed during this study are included in this published article [and its supplementary information file].
