# Supplementary material for: The prevalence of hyperglycemia and its association with perioperative outcomes in gynecologic surgery: a retrospective cohort study
Source: Perioper Med (Lond). 2023 Jun 2;12:19. doi: 10.1186/s13741-023-00307-1 (PMC10236760; doi:10.1186/s13741-023-00307-1)
Supplement: Supplementary file 2 — Additional file 2: Supplemental Table S1. Summary of complications, overall and by hyperglycemia status. Supplemental Table S2. Associations estimated from univariate analysis. [file 13741_2023_307_MOESM2_ESM.docx]

Supplemental Table S1. Summary of complications, overall and by hyperglycemia status.

| **Complication^a^** | **Overall**  ***(n=913)*** | **Hyperglycemia^b^** | | ***P^c^*** |
| --- | --- | --- | --- | --- |
|  |  | **No**  ***(n=846)*** | **Yes**  ***(n=67)*** |  |
| **Any Complication** | 242 (26.5%) | 217 (25.7%) | 25 (37.3%) | .04 |
| **Intraoperative** | 45 (4.9%) | 39 (4.6%) | 6 (9.0%) | .28 |
| **Perioperative Transfusion** | 45 (4.9%) | 40 (4.7%) | 5 (7.5%) | .32 |
| **Postoperative** |  |  |  |  |
| Wound | 49 (5.4%) | 43 (5.1%) | 6 (9.0%) | .17 |
| Respiratory | 14 (1.5%) | 11 (1.3%) | 3 (4.5%) | .04 |
| Cardiovascular | 17 (1.9%) | 15 (1.8%) | 2 (3.0%) | .48 |
| Gastrointestinal | 38 (4.2%) | 36 (4.3%) | 2 (3.0%) | .62 |
| Nervous System^d^ | 18 (2.0%) | 17 (2.0%) | 1 (1.5%) | .51 |
| Renal | 85 (9.3%) | 76 (9.0%) | 9 (13.4%) | .23 |
| Endocrine | 11 (1.2%) | 3 (0.4%) | 8 (11.9%) | <.001 |
| Sepsis | 4 (0.3%) | 4 (0.5%) | 0 (0.0%) | .57 |
| Venous Thromboembolism^d^ | 6 (0.7%) | 5 (0.6%) | 1 (1.5%) | .15 |

^a^ See Methods section for detailed list of complications in each category. Data reported as n (%).

^b^ Defined as blood glucose ≥ 140 g/dL

^c^ Determined by Pearson’s Chi-squared test.

^d^ Not including missing data on 13 patients for Nervous System, and 3 patients for Venous Thromboembolism.

Supplemental Table S2. Associations estimated from univariate analysis.

|  | **Hyperglycemia^a^** | | **Composite Complication^a^** | | **Wound Complication^a^** | |
| --- | --- | --- | --- | --- | --- | --- |
|  | ***(Events = 67)*** | | ***(Events = 242)*** | | ***(Events = 49)*** | |
| **Patient Characteristics** | **OR (95% CI)** | ***P*** | **OR (95% CI)** | ***P*** | **OR (95% CI)** | ***P*** |
| Age | 1.4 (1.2-1.7) | <0.001 | 1.2 (1.0-1.3) | .005 | 1.0 (0.8-1.2) | .94 |
| BMI, kg/m^2^ |  |  |  |  |  |  |
| < 25 | reference |  | reference |  | reference |  |
| 25-29.9 | 1.4 (0.7-3.1) | .37 | 0.9 (0.6-1.4) | .69 | 1.2 (0.5-2.7) | .71 |
| 30-34.9 | 1.0 (0.4-2.5) | .98 | 0.9 (0.5-1.4) | .53 | 0.4 (0.1-1.4) | .18 |
| 35-39.9 | 2.1 (0.9-4.9) | .08 | 1.0 (0.6-1.6) | .90 | 2.0 (0.8-4.9) | .13 |
| ≥40 | 2.8 (1.2-6.5) | .02 | 1.4 (0.9-2.4) | .17 | 2.0 (0.8-5.3) | .14 |
| Race |  |  |  |  |  |  |
| White | reference |  | reference |  | reference |  |
| Non-White | 1.0 (0.5-1.8) | .89 | 0.8 (0.6-1.3) | .51 | 0.7 (0.3-1.5) | .30 |
| Ethnicity |  |  |  |  |  |  |
| Non-Hispanic | reference |  | reference |  | reference |  |
| Hispanic | 0.6 (0.2-2.1) | .46 | 1.3 (0.7-2.2) | .40 | 1.3 (0.4-3.6) | .67 |
| Smoking status |  |  |  |  |  |  |
| Never | reference |  | reference |  | reference |  |
| Current | 0.4 (0.1-1.4) | .17 | 0.9 (0.6-1.6) | .81 | 0.7 (0.2-2.3) | .56 |
| Former | 1.0 (0.6-1.8) | .99 | 1.2 (0.8-1.7) | .37 | 1.5 (0.8-2.8) | .22 |
| Insurance status |  |  |  |  |  |  |
| Private | reference |  | reference |  | reference |  |
| Public | 1.3 (0.8-2.2) | .29 | 1.6 (1.1-2.1) | .006 | 1.6 (0.9-2.9) | .12 |
| Other | 0.8 (0.3-2.3) | .67 | 1.8 (1.1-3.0) | .03 | 0.6 (0.1-2.6) | .48 |
| ASA class |  |  |  |  |  |  |
| I-II | reference |  | reference |  | reference |  |
| ≥III | 3.7 (2.0-7.0) | <0.001 | 1.3 (1.0-1.8) | .06 | 1.4 (0.8-2.6) | .24 |
| Malignancy | 3.2 (1.9-5.4) | <0.001 | 2.4 (1.7-3.4) | <0.001 | 1.9 (1.0-3.7) | .05 |
| Cardiovascular disease | 3.9 (2.2-7.1) | <0.001 | 1.3 (0.9-1.7) | .13 | 1.4 (0.8-2.5) | .25 |
| Respiratory disease | 0.6 (0.3-1.3) | .18 | 0.6 (0.4-0.9) | .01 | 0.7 (0.3-1.7) | .42 |
| Diabetes | 29.3 (16.0-53.7) | <0.001 | 1.5 (1.0-2.2) | .05 | 1.7 (0.9-3.5) | .12 |
| Preoperative Hgb | 0.9 (0.8-1.0) | .08 | 0.9 (0.8-1.0) | .003 | 1.0 (0.8-1.2) | .99 |
| Case timing |  |  |  |  |  |  |
| First | reference |  | reference |  | reference |  |
| Not First | 0.8 (0.5-1.3) | .42 | 0.9 (0.7-1.2) | .43 | 0.7 (0.4-1.3) | .30 |
| Preoperative nurse visit | 0.9 (0.4-2.1) | .87 | 0.6 (0.4-1.0) | .04 | 0.6 (0.3-1.5) | .29 |
| Preoperative anesthesia visit | 0.5 (0.2-1.3) | .17 | 0.4 (0.2-0.8) | .01 | 0.3 (0.1-0.9) | .04 |
| Hyperglycemia status |  |  |  |  |  |  |
| No |  |  | reference |  | reference |  |
| Yes |  |  | 1.7 (1.0-2.9) | 0.04 | 1.8 (0.8-4.5) | .18 |
| Surgical Approach | N/A^b^ |  |  |  |  |  |
| MIS |  |  | reference |  | reference |  |
| Laparotomy |  |  | 3.7 (2.7-5.2) | <0.001 | 2.7 (1.5-4.9) | .001 |
| Division |  |  |  |  |  |  |
| General Gyn |  |  | reference |  | reference |  |
| MIGS |  |  | 1.0 (0.5-1.8) | .99 | 2.7 (0.6-11.8) | .19 |
| Urogynecology |  |  | 1.5 (0.8-2.6) | .19 | 1.8 (0.4-8.2) | .46 |
| Gyn Oncology |  |  | 2.8 (1.6-5.0) | .001 | 4.3 (1.0-18.7) | .06 |
| Operative time, per 60 minutes |  |  | 1.5 (1.4-1.7) | <0.001 | 1.2 (1.0-1.4) | .06 |
| Upper quartile EBL |  |  | 3.2 (2.4-4.5) | <0.001 | 1.6 (0.8-2.9) | .13 |

ASA, American Society of Anesthesiologists; BMI, body mass index; EBL, estimated blood loss; Gyn, gynecology; Hgb, hemoglobin in g/dL; MIGS, minimally invasive gynecologic surgery division; MIS, minimally invasive surgical route; N/A, not applicable

^a^ See Methods section for definitions.

^b^ Intraoperative independent variables not included in regression of preoperative hyperglycemia.
